# Effect of High- versus Low-Fat Meal on Serum 25-Hydroxyvitamin D Levels after a Single Oral Dose of Vitamin D: A Single-Blind, Parallel, Randomized Trial

**DOI:** 10.1155/2011/809069

**Published:** 2011-12-07

**Authors:** Fabiana Viegas Raimundo, Gustavo Adolpho Moreira Faulhaber, Paula Kalinka Menegatti, Leonardo da Silva Marques, Tania Weber Furlanetto

**Affiliations:** ^1^Programa de Pós-Graduação em Medicina: Ciências Médicas, Universidade Federal do Rio Grande do Sul, Rua Ramiro Barcelos 2400, 90035-003 Porto Alegre, RS, Brazil; ^2^Serviço de Medicina Interna, Hospital de Clínicas de Porto Alegre, Rua Ramiro Barcelos 2350/700, 90035-903 Porto Alegre, RS, Brazil

## Abstract

*Background/Aims*. Vitamin D_3_ is liposoluble, so dietary fat could increase its oral absorption. Our aim was to compare serum 25-hydroxyvitamin D [25(OH)D] after the oral intake of cholecalciferol with a high- or low-fat meal. *Methods*. In a single-blind, parallel clinical trial, 32 healthy physicians were divided into two groups. In the same day, they ingested 50,000 IU (1.25 mg) of vitamin D_3_ with food: group 1 (G1): lipids: 25.6 g and group 2 (G2) lipids: 1.7 g. Serum 25(OH)D (0, 7, and 14 days), and parathyroid hormone (PTH), and calcium (0 and 14 days) were measured. *Results*. Baseline mean serum 25(OH)D levels were 42.7 ± 19.0 nmol/L in G1 and 36.4 ± 19.0 nmol/L in G2 (*P* = 0.38). After cholecalciferol, mean serum 25(OH)D was higher in G1 (*P* < 0.001): 7 days: G1 = 46.2 (38.4–53.9) nmol/L and G2 = 33.7 (25.4–40.1) nmol/L; 14 days: G1 = 53.7 (45.2–62.1) nmol/L and G2 = 33.7 (25.2–42.2) nmol/L. Serum PTH and 25(OH)D were negatively correlated before and after the intake of vitamin D_3_, respectively, *r* = −0.42 (*P* = 0.02) and *r* = −0.52 (*P* = 0.003). *Conclusions*. A high-fat meal increased the absorption of vitamin D_3_, as measured by serum 25(OH)D.

## 1. Introduction

Vitamin D is important for human health [1], and lower serum 25-hydroxyvitamin D [25(OH)D] has been associated with increased mortality [2]. Moreover, a high prevalence of vitamin D deficiency has been identified worldwide in recent years [3]. Dietary supplements are useful to prevent and treat this deficiency [4].

As vitamin D is liposoluble, its oral absorption could increase if ingested with a fat-rich meal. Although there are several studies about the effect of different ways to supply vitamin D in its serum levels [5–9] or in serum 25(OH)D levels [10–14], only a few of these studies describe the amount of fat ingested with vitamin D [7, 9, 11]. Therefore, the aim of this study was to compare serum 25(OH)D levels after the oral intake of cholecalciferol with a high- or low-fat meal in young adults.

## 2. Materials and Methods

This single-blind parallel randomized trial included 32 healthy resident physicians in Porto Alegre, latitude 30°, Brazil. Height and weight were measured to calculate body mass index (BMI: weight (kg)/height^2^ (m)). Two different groups were formed with 16 individuals, each according to sex and BMI, and randomly assigned to a high-(Group 1: G1) or low-fat meal (Group 2: G2). The exclusion criteria were not drinking milk; BMI ≥30 kg/m^2^ or <18.5 kg/m^2^; known liver, kidney or endocrine disease; use of supplements of calcium and/or vitamin D; use of anticonvulsants, barbiturates, or glucocorticoids, and travel outside the Brazilian South region during the previous 120 days. Skin phototype was evaluated according to Fitzpatrick [15].

The low-fat meal contained skim milk, white bread with fruit jelly, and fruit salad. The high-fat meal contained whole milk, white bread with bologna, and vegetable oil margarine.

All participants came to the research unit on the same day at 7:00 AM, after overnight fasting. A blood sample was collected to measure 25(OH)D, parathyroid hormone (PTH), total calcium, albumin, magnesium, and creatinine, and a urine sample was collected to measure creatinine, calcium, and magnesium. Then, 50,000 IU of cholecalciferol in a capsule were administered by the oral route to each subject during a meal with high- or low-fat content (Table 1). The subjects ingested the whole content of the meal, and then were advised to avoid sun exposure and changes in their usual eating pattern for the next two weeks. The researchers who collected the samples were blinded to the groups.

Overnight fasting blood samples were collected after 7 days for measurement of 25(OH)D and 14 days for measurement of 25(OH)D, PTH, and total calcium. Serum was kept at −70°C until the assay of 25(OH)D and PTH. All samples were analyzed in the same run: 25(OH)D by chemiluminescence (LIAISON, DiaSorin Inc., Stillwater/MN, USA, intra-assay coefficient of variation = 5.5%) and PTH by electrochemiluminescence (Roche Diagnostics, Indianapolis/IN, USA, intra-assay coefficient of variation = 2.8%).

The number of subjects, calculated to detect a 30% difference in mean serum 25(OH)D levels between groups, with a standard deviation of 7.7 ng/mL [17], power of 80%, and *P* < 0.05, was 15 per group. One additional subject was included in each group to allow for losses.

Repeated measures ANOVA was used to compare mean serum 25(OH)D levels. Correlations were analyzed by the Pearson correlation coefficient. All data were analyzed with SPSSv.16.0, and differences were considered significant when *P* < 0.05.

Cholecalciferol in powder (Taizhou Hisound Chemical Co. Ltd, Taizhou, Zhejiang, China) was provided by DEG Ativando Princípios Company, São Paulo, SP, Brazil, which evaluated its content through HPLC (99.9%). The content of vitamin D_3_ in the capsules ranged from 48,170–52,777 IU. All of the vitamin D capsules contained lactose (82 mg) and crystalline micro cellulose (18 mg). One International Unit (1 IU) of vitamin D is equal to 0.025 micrograms.

The study was approved by the Ethics Committee of HCPA, and participants were included after written informed consent.

## 3. Results

**Figure 1:**
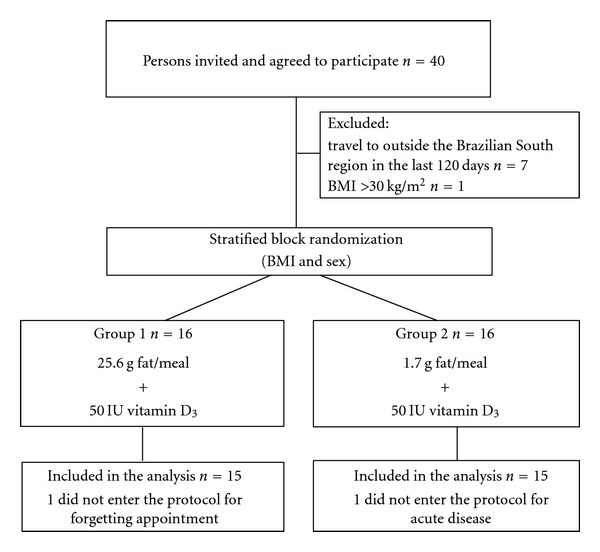
Flow diagram of the participants. Abbreviation: BMI-Weight (kg)/Height (m)^2^.

Forty resident physicians were invited to participate, and all agreed. Seven were excluded because they had traveled to regions with high UVB incidence in the last 120 days, and one was excluded for having BMI >30 kg/m^2^. Two participants, one from each group, did not enter the protocol, for missing the first appointment: one forgot and the other was acutely ill. All others were included in the analyses (Figure 1). The study was conducted in October 2009.

**Figure 2:**
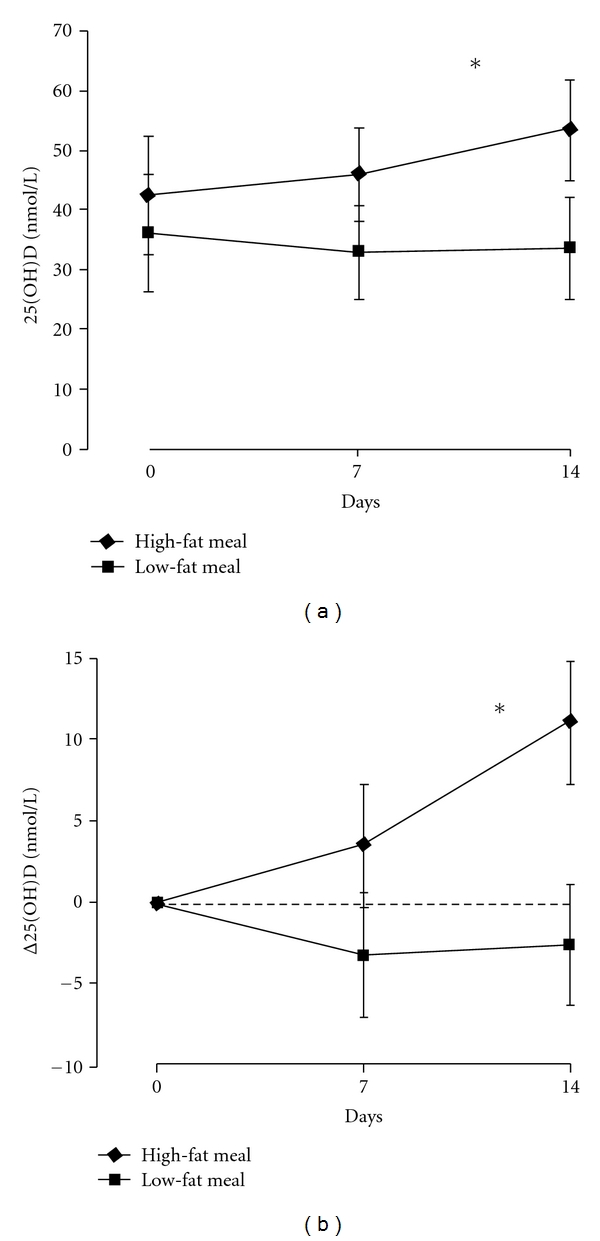
Mean serum 25(OH)D levels (a) and mean variation of serum 25(OH)D levels (b) were higher, after the intake of 50,000 IU of vitamin D_3_ with a high-fat meal (*n* = 15) when compared to a low-fat meal (*n* = 15). *P < 0.001, comparing the two slopes. Data are shown as mean and 95% confidence interval.

 The baseline characteristics are shown in Table 2. Phototypes ranged from I to II. Serum 25(OH)D was <75 nmol/L L in 28 subjects, <50 nmol/L in 21 subjects, and <25 nmol/L in 5 subjects. After cholecalciferol, mean serum 25(OH)D levels and mean variation of serum 25(OH)D levels were higher in G1 (*P* < 0.001), and the differences were significant at day 14 (Figures 2(a) and 2(b)). 

**Figure 3:**
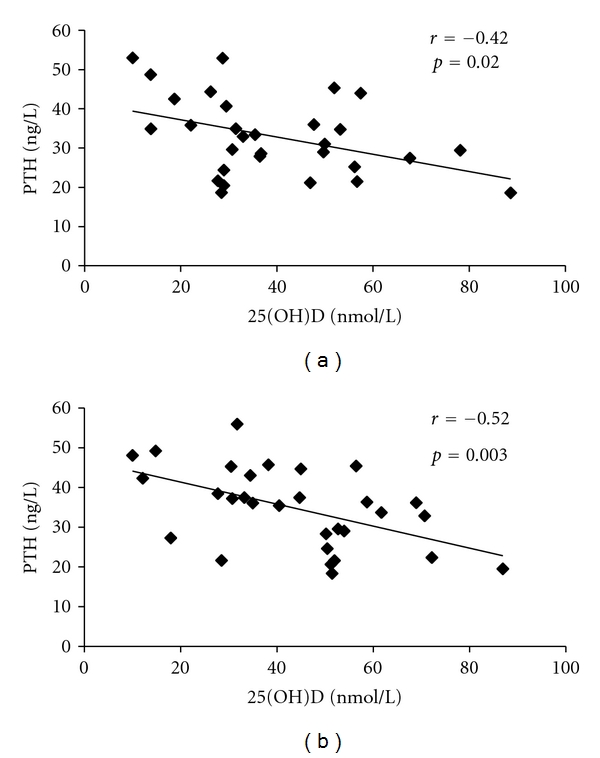
Correlation between serum parathyroid hormone (PTH) and 25-hydroxyvitamin D [25(OH)D] levels, in young adults (*n* = 30), at baseline (a), and 14 days after the oral intake of 50,000 IU of vitamin D_3_ (b).

 At day 14, no one had hypercalcemia, and mean serum total calcium (G1: 2.3 ± 0.1 mmol/L and G2: 2.2 ± 0.1 mmol/L; *P* = 0.11) and PTH (G1: 33.1 ± 11.0 ng/L; G2: 36.4 ± 9.0 ng/L, *P* = 0.37) were similar in both groups; the mean change in serum PTH was negative in G1 (−1.8 ± 7.2 ng/L) and positive (5.5 ± 7.1 ng/L) in G2 (*P* < 0.01). In all participants, serum PTH and 25(OH)D levels were negatively correlated at baseline and 14 days after the intake of vitamin D, as shown in Figure 3.

## 4. Discussion

The intake of 50,000 IU of cholecalciferol with a fat-rich meal increased mean serum 25(OH)D levels, in young adults. A change in mean serum 25(OH)D levels was not observed in the group that received vitamin D along with low-fat food, and its increase in the group that received the high-fat meal was small. As a placebo group was not studied, it is possible that serum 25(OH)D levels were decreasing in the sample studied due to the season [18].

The improved bioavailability of cholecalciferol when administered with fat-rich food could be due to a higher release of bile, allowing an increased incorporation of fat in the bile salt micelle, and its absorption [19]. Few studies have evaluated the effect of dietary fat in the absorption of vitamin D. In a clinical trial that compared the effect of 25,000 IU of vitamin D_2_ intake with whole milk (8.4 g of fat/serving), skimmed milk (2.4 g of fat/serving), or toast with 0.1 mL of corn oil, there was no difference in mean serum vitamin D_2_ levels, which suggested that its absorption is not dependent on fat [9]. Nevertheless, the content of fat ingested was small in all groups, so serum vitamin D levels could have been higher if the vitamin had been ingested with more fat. In another study, normal individuals consumed low-fat cheese (*∼*3 g of fat/serving) or high-fat cheese (*∼*11 g of fat/serving), fortified with 28,000 IU of vitamin D, or the same amount of vitamin D dissolved in ethanol, with or without food, per week, for 8 weeks. Mean serum 25(OH)D were similar in all groups treated with vitamin D and higher than placebo [14]. However, in these studies, the difference in the amount of fat provided by the meals for different groups was smaller than the one in the present study, and the total amount of fat offered in the meals was also smaller. Moreover, dissolving vitamin D in ethanol could have made its absorption easier.

In another study, higher mean serum vitamin D_2_ levels, in young and older adults, were observed when the vitamin was ingested with cheese (*∼*20 g of fat/serving) when compared to water, suggesting that food facilitates its absorption, and is not affected by age [7]. In this study, the fat content of cheese was similar to the one provided in our study. A recent uncontrolled study found higher mean serum 25(OH)D levels when vitamin D supplements were ingested during the largest meal of the day; however, the content of these meals was not described [20].

Although baseline mean serum 25(OH)D level was low in all subjects and 5/30 subjects had serum 25(OH)D level <25 nmol/L, serum PTH levels were within the normal range in all subjects. However, it is possible that at least part of the subjects had secondary hyperparathyroidism, as there was a negative correlation between serum PTH and 25(OH)D levels [21]. It is known that serum PTH levels increase with age, BMI, and low serum 25(OH)D levels [21, 22]. This study was made in spring, when lower serum 25(OH)D levels are expected in this region [18].

Our study has limitations. The absorption of vitamin D was evaluated with only two meals with highly different fat content; however, these are not unusual dietary patterns in everyday life. Moreover, as the two meals were different also in other components, to offer a similar amount of calories, it cannot be excluded that vitamin D_3_ absorption was decreased in the low-fat meal group, due to its higher fiber content. Also, it cannot be excluded that different amounts of vitamin D were ingested by the subjects, as the amount of vitamin D in food was not evaluated. Nevertheless, it is very improbable that dietary vitamin D influenced the results, because natural food contains only small amounts of vitamin D. In addition, the participants were advised to keep their usual dietary patterns until completing the study. Another limitation was the measurement of serum 25(OH)D levels as a surrogate for serum vitamin D levels. Nonetheless, this metabolite increases rapidly after vitamin D supplementation, and it is measured to evaluate vitamin D status and the effect of vitamin D supplementation [17, 23].

As vitamin D absorption could explain in part the variability in serum 25(OH)D levels after the oral supplementation of this vitamin, other studies should be designed to clarify the role of food and dietary fat in vitamin D absorption, allowing us to determine the most effective way to improve vitamin D nutrition.

In conclusion, the results of this small randomized controlled trial show that vitamin D supplementation is more effective when given with fat-containing food. These findings can have important implications to define the adequate dietary intake of vitamin D.

## Figures and Tables

**Table 1:** Nutritional composition of meals.^1^

Nutrients^2^	Group 1	Group 2
Lipids (%)	25.6 g (48.7)	1.7 g (3.3)
Carbohydrates (%)	43.3 g (36.6)	95.5 g (82.2)
Proteins (%)	17.2 g (14.5)	16.8 g (14.4)
Fiber	1,5 g	3,5 g
Energy	473 kcal	465 kcal

^1^According to the Brazilian Table of Food Composition [16].

^2^Percentage of total calories in the meal.

**Table 2:** Baseline characteristics of the study groups.

Parameters	Normal Range	Group 1 (*n* = 15)	Group 2 (*n* = 15)
Males/females (*n*)		6/9	6/9
Age (yr)		27.5 ± 2.0	26.7 ± 1.7
BMI (kg/m^2^)		22.3 ± 2.8	22.5 ± 2.6
Serum			
25(OH)D (nmol/L)		42,7 ± 19,0	36,4 ± 19,0
PTH (ng/L)	14.0–72.0	34.9 ± 9.9	31.0 ± 9.9
Albumin (g/L)	34.0–48.0	46.0 ± 3.0	46.0 ± 3.0
Calcium (mmol/L)	2.1–2.5	2.3 ± 0.1	2.3 ± 0.1
Creatinine (*μ*mol/L)	44.2–106.1	79.6 ± 17.7	79.6 ± 17.7
Magnesium (mmol/L)	0.7–1.1	0.9 ± 0.04	0.9 ± 0.04
Urine			
Creatinine (mmol/L)		17.92 ± 9.83	14.92 ± 5.31
Calcium (mmol/L)		2.8 ± 1.7	4.1 ± 2.4
Magnesium (mmol/L)		4.1 ± 1.6	4.3 ± 1.9

Data are shown as number of participants (*n*) or mean ± SD.

Abbreviations: BMI: body mass index; 25(OH)D: 25-hydroxyvitamin D; PTH: parathyroid hormone.
